# Supplementary material for: Effect of Silica-Based Nanomaterials on Seed Germination and Seedling Growth of Rice (Oryza sativa L.)
Source: Nanomaterials (Basel). 2022 Nov 24;12(23):4160. doi: 10.3390/nano12234160 (PMC9740595; doi:10.3390/nano12234160)
Supplement: Supplementary file 1 [file nanomaterials-12-04160-s001.zip › nanomaterials-2019588-supplementary.pdf]

Supporting Information

Effect of silica-based nanomaterials on seed germination and seedling growth of rice (*Oryza sativa* L.)

Yaqi Jiang [1], Jie Yang [1], Mingshu Li [1], Yuanbo Li [1], Pingfan Zhou [1], Quanlong Wang [1], Yi Sun [1], Guikai Zhu [1], Qibin Wang [1], Peng Zhang [2,3]*, Yukui Rui [1,4,5]* and Iseult Lynch [2]

[1] Beijing Key Laboratory of Farmland Soil Pollution Prevention and Remediation, College of Resources and Environmental Sciences, China Agricultural University, Beijing 100193, China

[2] Department of Chemistry, Queen Mary University of London, London E1 4NS, United Kingdom

[3] School of Geography, Earth and Environmental Sciences, University of Birmingham, Edgbaston, Birmingham B15 2TT, United Kingdom.;

[4] China Agricultural University Professor's Workstation of Yuhuangmiao Town, Shanghe County, Jinan, Shandong, China

[5] China Agricultural University Professor's Workstation of Sunji Town, Shanghe County, Jinan, Shandong, China

*Correspondence: ruiyukui@163.com (Y. R.); p.zhang.1@bham.ac.uk (P. Z.)

Supplementary information:

Number of pages: 4

Number of Supplementary Figures: 3

Number of Supplementary Tables: 1

Figure:

Figure S1TEM and SEM images of SiO_2 NMs(A, C) and SiC NMs(B,D)

Figure S2 FTIR spectra of SiO_2 NMs (A) and SiC NMs (B)

Figure S3 Images of rice leaves(A) and roots(B)

Table:

Table S1 The composition of Kimura nutrient solution

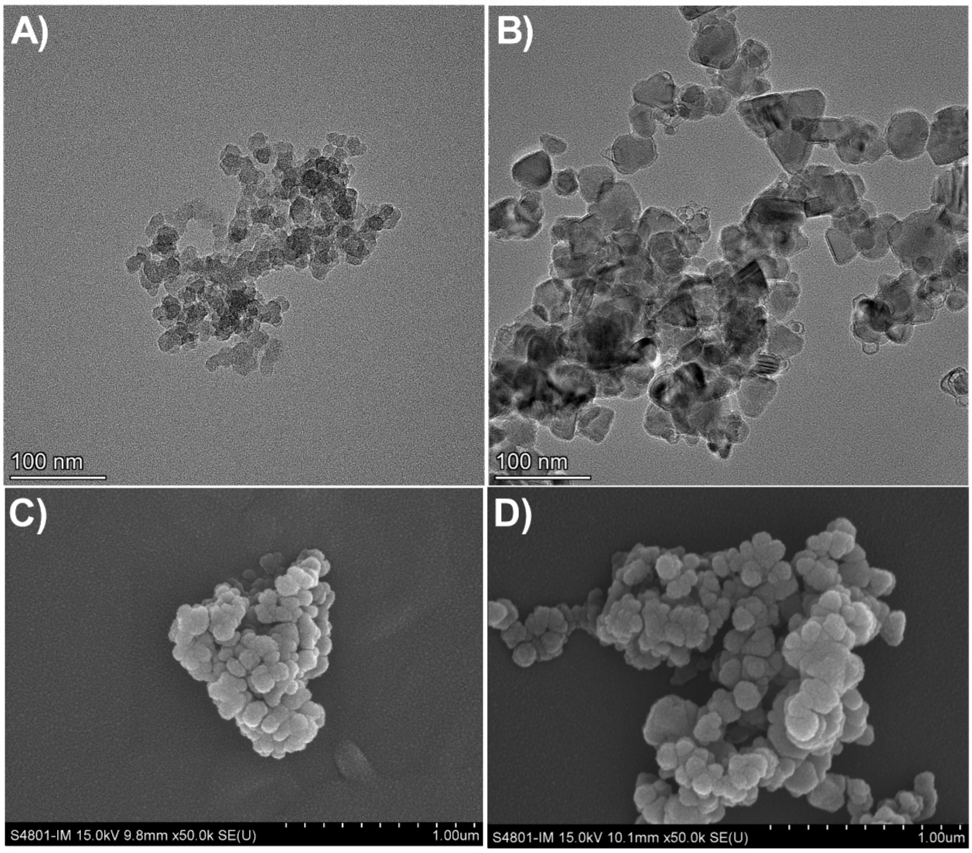

Figure S1 TEM and SEM images of SiO_2 NMs (A, C) and SiC NMs (B, D)

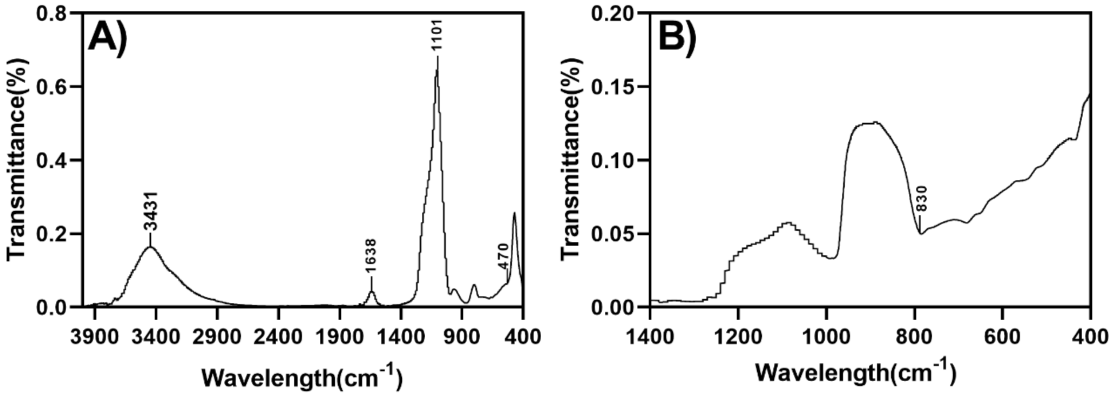

Figure S2 FTIR spectra of SiO_2 NMs (A) and SiC NMs (B)

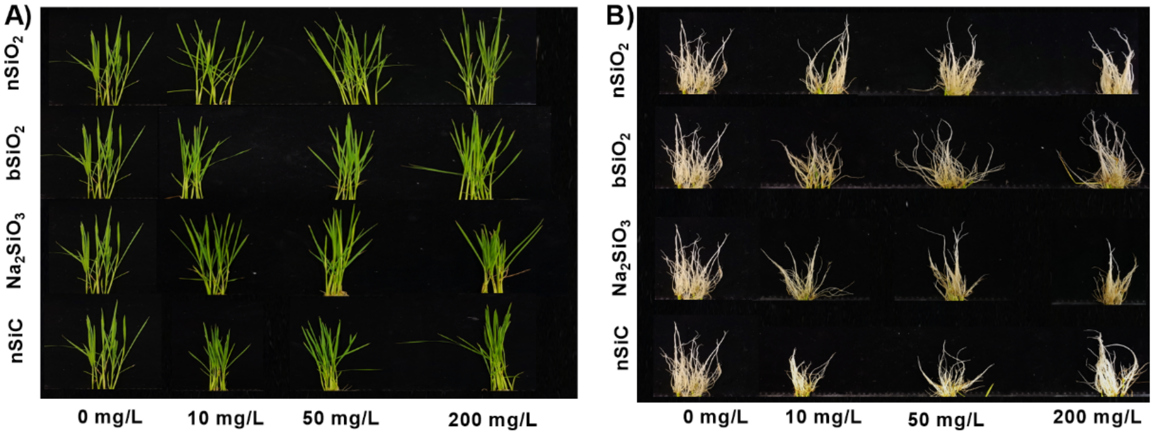

Figure S3 Images of rice leaves(A) and roots(B)

Table S1 The composition of Kimura nutrient solution

Chemicals	Concentrations
$Ca(NO_3)_2$	0.37 mM
KNO_3	0.18 mM
$(NH_4)_2SO_4$	0.37 mM
Fe(II)-EDTA	0.05 mM
$CuSO_4$	0.001 mM
NaCl	0.1 mM
H_3BO_4	0.01 mM
$MgSO_4$	0.55 mM
KH_2PO_4	0.18 mM
K_2SO_4	0.09 mM
$ZnSO_4$	0.001 mM
$MnSO_4$	0.005 mM
Na_2MoO_4	0.0005 mM
$CoSO_4$	0.0002 mM
